# Impact of health warning labels on snack selection: An online experimental study

**DOI:** 10.1016/j.appet.2020.104744

**Published:** 2020-11-01

**Authors:** Natasha Clarke, Emily Pechey, Eleni Mantzari, Anna K.M. Blackwell, Katie De-loyde, Richard W. Morris, Marcus R. Munafò, Theresa M. Marteau, Gareth J. Hollands

**Affiliations:** a Behaviour and Health Research Unit, Institute of Public Health, University of Cambridge, Cambridge, UK; b Tobacco and Alcohol Research Group, School of Psychological Science, University of Bristol, Bristol, UK; c Bristol Medical School, University of Bristol, Bristol, UK

**Keywords:** Health warning labels, Pictorial labels, Graphic warnings, Snacks, Food, Choice architecture, Energy-dense

## Abstract

Excessive consumption of energy-dense food increases the risk of obesity, which in turn increases the risk of non-communicable diseases, including heart disease, type 2 diabetes and most non-smoking-related cancers. Health warning labels (HWLs) that communicate the adverse health consequences of excess energy consumption could reduce intake of energy-dense foods. The aim of the current study was to estimate the impact on selection of energy-dense snacks of (a) image-and-text HWLs (b) text-only HWLs and (c) calorie information. In a between-subjects, 3 (HWL: image-and-text, text-only, no label) x 2 (calorie information: present, absent), factorial experimental design, participants (N = 4134) were randomised to view a selection of energy-dense and non-energy-dense snacks with one of five label types or no label. The primary outcome was the proportion of participants selecting an energy-dense snack in a hypothetical vending machine task. The proportion of participants selecting an energy-dense snack was reduced in all label groups, relative to the no label group (no label: 59%; calories only: 54%; text-only HWL: 48%; text-only HWL with calories: 44%; image-and-text HWL: 37%; image-and-text HWL with calories: 38%). Compared to the no label group, participants were least likely to select an energy-dense snack in the image-and-text HWL group (OR = 0.46, 95%CI = 0.40, 0.54, p < 0.001). Health warning labels – particularly those including an image and text - have the potential to reduce selection of energy-dense snacks in an online setting. Their impact on selection and consumption in real-world settings awaits testing.

## 1 Introduction

The prevalence of obesity worldwide has nearly tripled since 1975 (WHO, 2018b). In the UK, 61% of adults and 29% of children were classified as overweight or obese in 2016 (HSCIC, 2017). Excess weight is a risk factor for several diseases, with obesity being the second most preventable cause of cancer after smoking, and increasing the risk of heart disease and type 2 diabetes (Brown et al., 2018; Wang, McPherson, Marsh, Gortmaker, & Brown, 2011). A key cause of rising obesity rates is the increase in consumption of excess energy from food, particularly from high fat, energy-dense and nutrient poor foods (WHO, 2018b), driven by the obesogenic environment (Brandkvist et al., 2019). Multiple interventions are needed that are capable of shifting behaviour at population level, including those that target the immediate physical environments that cue much consumption (Hollands et al., 2017). One potential intervention to discourage consumption of energy-dense products is through altering the ways in which products are labelled.

Health warning labels (HWLs) are currently mandated for use on tobacco packaging in many countries including the UK, Australia and Canada (CCS, 2018). There is a substantial body of evidence demonstrating their impact on a range of outcomes including cessation-related behaviours (Hammond, 2011), with evidence indicating labels that generate negative emotions are most effective (Cho et al., 2018). This is shown by a greater effect of image-and-text (often called ‘pictorial’ or ‘graphic’) warnings than text-only warnings (Brewer et al., 2016; Hammond, 2011; Noar et al., 2015), including in socially and materially deprived groups in whom smoking rates are frequently higher (Thrasher et al., 2012). Given that the current implementation of HWLs on tobacco provides clear evidence that they are a feasible population-level intervention, there is high interest amongst researchers, policy-makers and the general media (Al-Hamdani & Smith, 2017; Parry, 2014; Popova, 2016) in their possible application to other health-damaging products including some foods, alcoholic and non-alcoholic drinks. Eating and drinking behaviours are not directly comparable to smoking behaviours, therefore direct evidence in these specific contexts is needed for the potential impact of HWLs on the consumption of these products before their implementation can be considered.

There is a near-complete absence of evidence of the effect of HWLs beyond tobacco, with only a small number of studies conducted to date (for review, see Clarke et al., 2020b). Most food studies have focused on sugar sweetened beverages (SSBs). These suggest that text-only HWLs decrease the likelihood of buying SSBs (Bollard, Maubach, Walker, & Ni Mhurchu, 2016), and image-and-text HWLs reduce intentions to purchase, preferences for and hypothetical selection of SSBs (Adams, Hart, Gilmer, Lloyd-Richardson, & Burton, 2014; Bollard et al., 2016; Mantzari, Vasiljevic, Turney, Pilling, & Marteau, 2018), as well as real-life purchases (Donnelly, Zatz, Svirsky, & John, 2018; Grummon et al., 2019). Reducing the energy consumed from energy-dense snack foods – which tend to have limited nutritional value (Dunford & Popkin, 2017) – is another relevant public health target, given adults on average consume 200 kcal per day over their recommended energy intake in the UK (PHE, 2018). There are a small number of studies suggesting warnings on snack foods reduce purchasing, intentions to consume, and increase dietary control and motivation to change eating behaviour (Acton, Jones, Kirkpatrick, Roberto, & Hammond, 2019; David et al., 2018; Neal et al., 2017; Rosenblatt, Dixon, Wakefield, & Bode, 2019; Rosenblatt et al., 2018a, 2018b). Further research is needed to examine the potential impact of food HWLs on behaviour and to elucidate the types of HWLs – such as text-only vs image-and-text or labels illustrating different consequences – likely to be most effective.

The potential for HWLs to increase defensive reactions and their acceptability are also important considerations alongside effectiveness. Tobacco research shows that HWLs can increase reactance and avoidance behaviours (*i.e.* annoyance at or deliberately not engaging with the HWL) (Maynard et al., 2014; McCloud, Okechukwu, Sorensen, & Viswanath, 2017), although these behaviours do not necessarily interfere with quitting behaviours (Brewer et al., 2019; Cho et al., 2016). HWLs placed on alcoholic beverages that include shocking or explicit pictures are rated as more effective than those with less severe pictures (Maynard, Gove, Skinner, & Munafò, 2018), but have been shown to increase reactance and avoidance behaviours (Sillero-Rejon et al., 2018) and may be less acceptable (Clarke et al., 2020b; Pechey et al., 2020). Initial studies suggest that text-only HWLs on SSBs (Roberto, Wong, Musicus, & Hammond, 2016) and image-and-text HWLs on energy-dense snacks are generally accepted (Pechey et al., 2020). Evidence also suggests that communicating the effectiveness of HWLs may increase policy support (Donnelly et al., 2018), a finding that aligns with results of a recent meta-analysis (Reynolds, Stautz, Pilling, Linden, & Marteau, 2020).

Calorie information on labels may also reduce the selection and consumption of energy (Ares et al., 2018; Crockett et al., 2018; Ni Mhurchu, Eyles, Jiang, & Blakely, 2018), but their additive impact with HWLs is unknown. Calorie information is currently available on the back of packaging on most foods in jurisdictions where this is mandated as part of food labelling policies. Although rarely mandatory, calorie information may also be displayed on front-of-package (FOP) as part of voluntary schemes. For example, in the UK it is presented on the majority (60%) of the packaged food and drink market (WHO, 2018a). Therefore, if HWL labels were implemented they would likely be presented alongside calorie information. By contrast, HWLs depicting the consequences of excessive calorie consumption would most plausibly be placed only on more energy-dense products.

The primary aim of the current study was to assess the impact on selection of energy-dense snacks of: (a) HWLs communicating the adverse health consequences of excess energy consumption placed on energy-dense snacks, presented as text, with and without images, and (b) labels communicating energy content (calorie information) placed on all snacks. It was hypothesised that image-and-text and text-only HWLs placed on energy-dense snacks and calorie content labels placed on all snacks would decrease the selection of energy-dense snacks. Secondary aims were to assess the impact of HWLs on emotional and cognitive responses - including negative emotional arousal, reactance, avoidance, and acceptability.

## 2 Methods

The study protocol and a detailed analysis plan were pre-registered on the Open Science Framework (https://osf.io/k7tw5/).

### 2.1 Design

The study was conducted on the online survey platform Qualtrics, using a between-subjects factorial experimental design with six conditions: 3 (HWL: text-only, image-and-text, no HWL) x 2 (calorie information: present vs absent) (Box 1).

Box 1 **Study design.**

**Figure:**
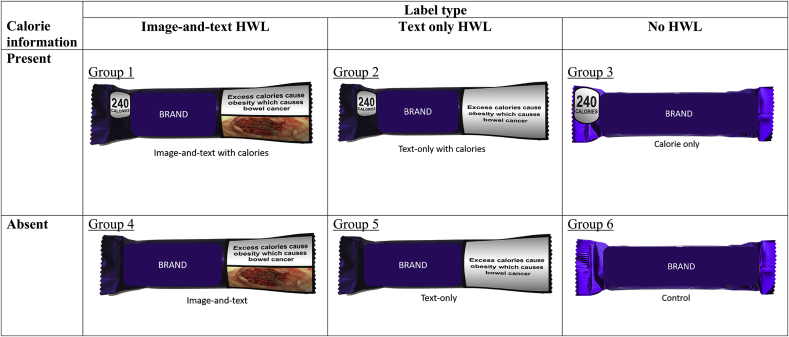

Alt-text: Box 1

### 2.2 Participants

Those eligible to participate were aged over 18 and regularly consumed energy-dense snacks – such as biscuits, cakes, chocolate, or crisps, at least one a week (participants were asked: “How often do you eat snacks from the following categories?” and indicated the frequency of which they consumed biscuits, cakes, chocolate, or crisps. Those who consumed one or more of these snacks at least once a week were eligible for inclusion). A general population sample, representative of the UK population, was recruited through a market research agency (Dynata). Individuals with a range of BMIs were included in the study as government guidance suggests eating less energy-dense food is suitable for most people in the UK, regardless of weight (BNF, 2016).

Based on a previous study assessing the impact on selection of warning labels on sugar sweetened beverages (Mantzari et al., 2018), the expected difference in the proportion of participants selecting an energy-dense snack between the different HWL types was 5.7 percentage points. In the current study, there were three HWL types: image-and-text HWL; text-only HWL; no HWL (Box 1). Each of the image-and-text (Groups 1 and 4) or text only HWL groups (Groups 2 and 5) was expected to show this difference when compared with no HWL (Groups 3 and 6). To detect these differences with 80% power and alpha of 0.025 (applying Bonferroni adjustment for 2 separate comparisons), it was calculated that 1360 participants for each of these 3 HWL groups was needed, giving a minimum sample size requirement of 4080. The sample of 4080 participants can be divided between those in groups with and without calorie information (Groups 1,2,3 vs Groups 4,5,6, respectively), allowing an independent comparison with more than 80% power with alpha of 0.05 to detect the same degree of difference (5.7%) for the calorie information being present or absent.

### 2.3 Interventions

#### 2.3.1 Label design

The specific adverse health consequences used for the image-and-text HWLs used in the study were selected based on the results of another study (Pechey, Jenkins, Cartwright, & Marteau, 2020, which aimed to identify the images eliciting the highest levels of negative emotional arousal. Given concerns about the potential weight stigma associated with images related to obesity (Hayward & Vartanian, 2019), the HWLs used were designed in line with published guidelines (EASO). In the control group, branded labels were displayed on the products in their original form. In the label groups, brand information was moved to remain clearly visible alongside the labels. The labels and label-product combinations used in the study were prepared by a graphic designer (see https://osf.io/kf3r7/for stimuli). The three HWLs selected for the main study depicted the links between calories, obesity and bowel cancer, heart disease and type 2 diabetes. Calorie information was given per package and was presented as black text on a white background. Further details on the selection process and label images can be found in the supplementary material (1).

Labelling is classed as an Information intervention within the TIPPME typology of such interventions (Hollands et al., 2017).

### 2.4 Measures

#### 2.4.1 Primary outcome

*Selection task*. The primary outcome was the proportion of participants selecting an energy-dense snack from a range of twelve branded snacks, selected for use in the study based on their energy-density and calorie content (six non-energy-dense; six energy-dense); *see procedure for details*.

#### 2.4.2 Secondary outcomes

*Negative emotional arousal*, assessed using a four-item measure (Cronbach's α = 0.91), previously used to assess the impact of warning labels on cigarette packages (Kees, Burton, Craig Andrews, & Kozup, 2010). Responses were denoted using seven-point scales: ‘How [afraid/worried/uncomfortable/disgusted] does the label on this snack make you feel?’ with scores ranging from 1 (not at all afraid/worried/uncomfortable/disgusted) to 7 (very afraid/worried/uncomfortable/disgusted). A mean average was calculated for the four items.

*Reactance and avoidance (defensive reactions)*, assessed using two separate items, previously used to assess the impact of warning labels on alcohol products (Blackwell, Drax, Attwood, Munafò, & Maynard, 2018). The items were taken from a 27-item scale developed for reactance to tobacco health warnings (Hall et al., 2016). Responses were denoted using seven-point scales: Reactance: ‘Are these labels annoying?‘; Avoidance: ‘Are you like to avoid these labels?‘, with scores ranging from 1 (not annoying/not at all) to 7 (extremely annoying/very likely). These items were scored separately.

*Perceived disease risk relating to consuming the energy-dense snack*, assessed using a three-item measure (Cronbach's α = 0.75) with each item assessed using a seven-point scale, and the mean average calculated, and adapted from a measure used to assess the impact of HWLs on SSBs (Roberto et al., 2016): ‘Consuming this snack often would [lead you to gain weight/increase your risk of [heart disease/cancer]/help you lead a healthier life]’, with scores ranging from 1 (strongly disagree) to 7 (strongly agree).

*Acceptability of health warning labels,* assessed using one item with responses denoted on a seven point scale, adapted from a similar measure used to assess the acceptability of a sugar tax (Reynolds, Pilling, & Marteau, 2018): ‘Do you support or oppose putting this label on high calorie snacks?‘, with scores ranging from 1 (strongly oppose) to 7 (strongly support). A score higher than the midpoint (four) was taken as indicating acceptability.

### 2.5 Other measures

*Demographic measures:* age, gender, ethnicity, education (highest level), self-reported weight and height (BMI calculated).

*Weekly snack consumption:* frequency of consumption of energy-dense snack foods from the following categories: biscuits and cookies, cakes, muffins or pastries, chocolate confectionery, sweet confectionery and crisps.

*Attention check:* ‘When did you last fly to Mars?’ Those not answering ‘never’ from a range of responses were screened out of the study.

### 2.6 Procedure

Ethical approval was granted by the Cambridge Psychology Research Ethics Committee (PRE.2018.071). After completing screening and demographic questions (age, gender, ethnicity and education) participants were randomised within the Qualtrics software to one of six experimental groups (Box 1). First, participants completed the selection task (primary outcome) and were shown a range of twelve branded snacks (six non-energy-dense: energy-density less than 3.5 calories per gram and less than 150 calories in total; six energy-dense: energy-density above 3.5 calories per gram and more than 200 calories in total). The energy-dense snack selection contained crisps, chocolate bars and chocolate and biscuit-based desserts. The non-energy-dense snack selection contained vegetable crisps, cereal bars, fruit and yoghurt. The products and the cut-offs are similar to those in comparable studies, in which foods were dichotomised into higher and lower calorie groups (Pechey, Cartwright, et al., 2019; Pechey, Jenkins, et al., 2019; Pechey & Marteau, 2018). All snacks were widely available in UK supermarkets (see supplementary material (S2) for details on snacks including brand and nutritional information). Participants viewed images of each of the 12 snacks in turn and then simultaneously viewed images of all the 12 snacks presented in randomised order, similar to a vending machine layout (for images of the snack selection see: https://osf.io/kf3r7/). Participants were given the following instructions: *“Imagine you are at a vending machine. Please select the snack you would like to eat right now.*” Each snack either displayed no calorie information or a calorie information label, and the energy-dense snacks had either no warning or a HWL. In the HWL groups, each energy-dense snack displayed one of three different HWLs, *i.e.*, one of the three health consequences, so that all health consequences were shown equally across the selection.

Second, participants viewed an image of a chocolate bar with or without a label depending on their allocated group, and completed the secondary outcome measures in the following order: negative emotional arousal, reactance, avoidance, perceived disease risk, acceptability. For the acceptability outcome only, participants in the control condition were re-randomised to one of the five label conditions.

Finally, participants completed further demographic questions: height, weight and energy-dense snack consumption frequency.

Participants could not proceed without answering all questions. Inattentive participants were screened out via the attention check embedded in the study and sampling continued until the quota was filled. All participants who successfully completed the study were debriefed and reimbursed for their participation. Data were collected in April 2019.

### 2.7 Statistical analysis

Descriptive statistics were used to compare baseline characteristics of those randomised to the six study groups to check for successful randomisation.

Logistic regressions were performed to assess the odds of selecting an energy-dense snack in each study group, using the ‘no label’ group as the reference category. A factorial 3 (HWL group) x 2 (calorie information) logistic regression model then assessed the impact of HWLs and calorie labels and the interaction between these.

For continuous secondary outcomes, normality was assessed, and an ANOVA (analysis of variance) was used to assess the differences between study groups, using the ‘no label’ group as the reference category. A 3 (HWL group) x 2 (calorie information) ANOVA model was then used to assess differences in all secondary outcomes (apart from acceptability, due to the re randomisation of the no label group) between HWLs and calorie labels and the interaction between these. Due to deviations from normality in the secondary outcomes, all analyses were repeated using a bootstrapping method and produced similar results. A detailed analysis plan was pre-registered and the full dataset are available (registration details: https://osf.io/zvrs5).^1^

## 3 Results

### 3.1 Participant characteristics

**Fig. 1:**
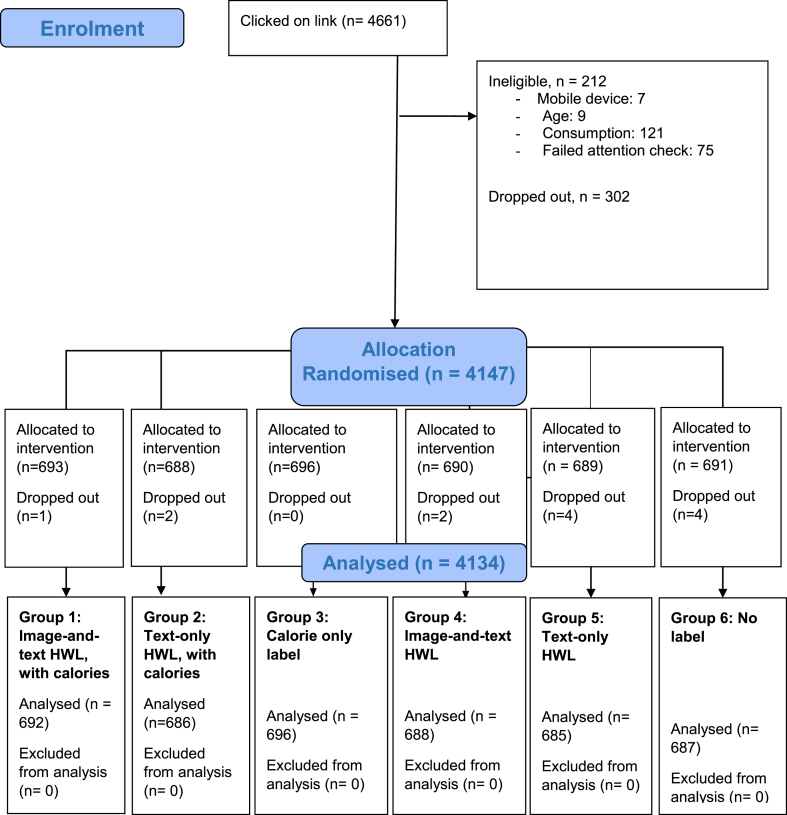
CONSORT flow diagram.

In total, 4147 participants were randomised and 4134 completed the study. Fig. 1 shows the flow of participants through the study. Participant characteristics are presented in Table 1. Just over 50% of the sample were female and the mean age was 47.2 (SD = 16.0). Groups were well balanced on all characteristics.

**Table 1:** Participant characteristics (n (%), unless otherwise stated).

	Calorie information					
	Present			Absent		
	Group 1: Image-and-text HWL n = 692	Group 2: Text-only HWL n = 686	Group 3: No HWL n = 696	Group 4: Image-and-text HWL n = 688	Group 5: Text-only HWL n = 685	Group 6: No label n = 687
**Snack consumption frequency (biscuits, cakes, chocolate, cookies, crisps)**						
Daily	473 (68%)	468 (68%)	455 (65%)	454 (66%)	462 (67%)	467 (68%)
Weekly	219 (32%)	218 (32%)	241 (35%)	234 (34%)	223 (33%)	220 (32%)

**BMI (mean** ± **SD)**	26.7 (5.8)	26.3 (6.2)	26.3 (6.2)	26.5 (5.4)	26.7 (6.3)	26.7 (5.9)

**Age (mean** ± **SD)**	47.6 (15.9)	47.2 (16.0)	47.2 (16.1)	47.0 (16.1)	46.5 (15.7)	47.8 (16.3)
**Gender**						
Male	337 (49%)	330 (48%)	335 (48%)	339 (49%)	334 (49%)	326 (47%)
Female	355 (51%)	354 (52%)	360 (52%)	346 (50%)	349 (51%)	361 (53%)
Other	0	1 (<1%)	1 (<1%)	2 (<1%)	1 (<1%)	0
Prefer not to say	0	1 (<1%)	0 (<1%)	1 (<1%)	1 (<1%)	0
**Ethnicity**						
White	631 (91%)	628 (92%)	641 (92%)	610 (89%)	612 (90%)	634 (92%)
Non-white	56 (8%)	55 (8%)	46 (7%)	71 (10%)	69 (10%)	51 (7%)
Prefer not to say	5 (1%)	3 (<1%)	9 (1%)	7 (1%)	4 (<1%)	2 (<1%)
**Education**						
No Bachelor's degree	354 (51%)	363 (53%)	333 (48%)	325 (47%)	327 (48%)	347 (51%)
Bachelor's degree or higher	338 (49%)	323 (47%)	363 (52%)	363 (53%)	358 (52%)	340 (49%)

*Standard deviation (SD). Body mass index (BMI)*. Health warning label (HWL). *Note:* Missing/prefer not to answer data is listed in the table but all % are valid %.

### 3.2 Primary outcome - energy-dense snack selection

Energy-dense snack selection was lower for all label types compared to no label (see Table 2). The mean number of calories selected in each group were, no label: 179 (SD = 98); calories only: 170 (SD = 99); text-only HWL: 160 (SD = 101); text-only HWL with calories: 148 (SD = 99); image-and-text HWL: 140 (SD = 94); image-and-text HWL with calories: 138 (SD = 92). Logistic regressions showed that compared to the no label group, all labels decreased the odds of selecting an energy-dense snack (see Table 3).

**Table 2:** **Primary (% (n)) and secondary outcomes (mean (**± **SD))**.

	Calorie information					
	Present			Absent		
	Group 1: Image-and-text HWL n = 692	Group 2: Text-only HWL n = 686	Group 3: No HWL n = 696	Group 4: Image-and-text HWL n = 688	Group 5: Text-only HWL n = 685	Group 6: No label n = 687
**Primary outcome**						
Proportion choosing energy-dense snack	38% (260)	44% (299)	54% (374)	37% (255)	48% (327)	59% (405)
	–					
**Secondary outcomes**						
Negative emotional arousal	4.19 (1.76)	3.57 (1.70)	2.43 (1.62)	4.18 (1.74)	3.44 (1.75)	1.62 (1.26)
Reactance	4.30 (2.11)	3.92 (2.04)	2.09 (1.60)	4.33 (2.03)	4.22 (2.05)	1.62 (1.30)
Avoidance	4.24 (2.06)	3.74 (1.96)	2.53 (1.84)	4.17 (2.04)	3.69 (1.90)	1.91 (1.62)
Perceived disease risk	20.84 (4.69)	20.68 (4.95)	19.71 (4.82)	20.94 (4.85)	20.37 (4.83)	19.43 (4.53)
Acceptability^a^	4.04 (2.00)	4.53 (1.87)	5.77 (1.41)	3.92 (2.05)	4.26 (1.87)	–

HWL = health warning label. SD = Standard deviation.

a Re-randomisation, into one of the other 5 groups, occurred for the no HWL group therefore the total n for this variable were: image-and-text with calorie n = 828, text-only with calorie n = 824, calorie information only n = 833, image-and-text n = 827, text-only n = 822.

**Table 3:** Odds Ratios (ORs) and Mean Differences (MDs) for the comparisons between label groups and the control group (no label), for primary and secondary outcomes.

	Calorie information					
	Present			Absent		
	Group 1: Image-and-text HWL n = 692	Group 2: Text-only HWL n = 686	Group 3: No HWL n = 696	Group 4: Image-and-text HWL n = 688	Group 5: Text-only HWL n = 685	Group 6: No label n = 687
**Primary outcomes (OR, 95% CI, *p* value)**						
Energy-dense snack selection	0.42 (0.34, 0.52) *p* < 0.001	0.54 (0.43, 0.67) *p* < 0.001	0.81 (0.65, 1.00) *p* = 0.051	0.41 (0.33, 0.51) *p* < 0.001	0.64 (0.51, 0.79) *p* < 0.001	–
**Secondary outcomes (MD, 95% CI, *p* value, effect size (Cohen's *d*))**						
Negative emotional arousal	2.57 (2.40, 2.75) *p* < 0.001, *d* = 1.68	1.95 (1.77, 2.12) *p* < 0.001, *d* = 1.30	0.81 (0.63, 0.98) *p* < 0.001, *d* = 0.56	2.56 (2.38, 2.73) *p* < 0.001, *d* = 1.69	1.82 (1.64, 1.99) *p* < 0.001, *d* = 1.19	–
Reactance	2.68 (2.48, 2.88) *p* < 0.001, *d* = 1.53	2.30 (2.10, 2.50) *p* < 0.001, *d* = 1.34	0.48 (0.28, 0.68) *p* < 0.001, *d* = 0.32	2.71 (2.52, 2.91) *p* < 0.001, *d* = 1.59	2.60 (2.40, 2.80) *p* < 0.001, *d* = 1.52	–
Avoidance	2.33 (2.13, 2.53) *p* < 0.001, *d* = 1.26	1.83 (1.63, 2.03) *p* < 0.001, *d* = 1.02	0.62 (0.42, 0.82) *p* < 0.001, *d* = 0.36	2.26 (2.06, 2.46) *p* < 0.001, *d* = 1.23	1.78 (1.57, 1.98) *p* < 0.001, *d* = 1.01	–
Perceived disease risk	1.41 (0.91, 1.92) *p* < 0.001, *d* = 0.31	1.25 (0.74, 1.76) *p* < 0.001, *d* = 0.26	0.28 (−0.23, 0.78) *p* = 0.280, *d* = 0.06	1.51 (1.00, 2.01) *p* < 0.001, *d* = 0.32	0.94 (0.44, 1.45) *p* < 0.001, *d* = 0.20	–
Acceptability^a^	−0.22 (−0.40, −0.04) *p* = 0.017, *d* = −0.11	0.27 (0.09, 0.45) *p* = 0.003, *d* = 0.14	1.51 (1.33, 1.69) *p* < 0.001, *d* = 0.91	−0.34 (−0.52, −0.16) *p* < 0.001, *d* = −0.17	–	–

HWL = health warning label, OR = odds ratio, CI = confidence interval, MD = mean difference.

a Re-randomisation, into one of the other 5 groups, occurred for the control group therefore the total n for this variable were: image-and-text with calorie n = 828, text-only with calorie n = 824, calorie information only n = 833, image-and-text n = 827, text-only n = 822, The reference group for this analysis was the image-and-text HWL, with calorie group.

In a factorial 3 (image-and-text vs. text-only vs. no label) x 2 (calorie information vs. no calorie information) model there was evidence of a main effect of image-and-text (OR [odds ratio] = 0.46, 95%CI [confidence interval] = 0.40, 0.54, p < 0.001) compared to no HWL, a main effect of text (OR = 0.65, 95%CI = 0.56, 0.76, p < 0.001) compared to no HWL, and a modest main effect of calorie information (OR = 0.89, 95%CI = 0.78, 1.00, p = 0.054) compared to no calorie information. There was no evidence of an interaction between HWL group and calorie information (p = 0.282). Overall, in this 3 × 2 design, image-and-text HWLs (37% selected an energy-dense snack) were more effective for decreasing energy-dense snack selection compared to text-only HWLs (46%), calorie information alone (54%) and no label (59%). When there was no HWL, the addition of calorie information increased effectiveness (5% decrease in energy-dense snack selection), although in the presence of a text-only HWL it had only a small additional impact (4% decrease) and no additional impact in the presence of an image-and-text HWL (1% increase).

### 3.3 Secondary outcomes (see Table 2 for full descriptive data and Table 3 for estimated effects)

#### 3.3.1 Negative emotional arousal

Compared to the no label group, all labels increased negative emotional arousal (all p < 0.001) (Table 3). The 3 × 2 ANOVA model indicated there was a main effect of HWL type (F (2, 4128) = 619.34, p < 0.001), with a larger increase in negative emotional arousal compared to no label in the image-and-text HWL groups (MD [mean difference] = 2.16, 95%CI = 2.04, 2.28, p < 0.001, d = 1.32), than in the text-only HWL groups (MD = 1.48, 95%CI = 1.36, 1.60, p < 0.001, d = 0.85). Adding calorie information also increased negative emotional arousal scores (F (1, 4128) = 38.35, p < 0.001), but only by a small amount (MD = 0.32 95%CI = 0.22, 0.42, p < 0.001, d = 0.17). There was a significant interaction between HWL group and calorie information (F (2, 4128) = 23.22, p < 0.001), indicating that calorie information on its own increased negative emotional arousal compared to when no label was present, increased negative emotional arousal in addition to a text-only HWL but had no additional impact to an image-and-text HWL.

#### 3.3.2 Reactance and avoidance

Compared to the no label group, all labels increased scores on reactance and avoidance (all p < 0.001) (Table 3). The 3 × 2 ANOVA model indicated there was a main effect of HWL type (reactance: F (2, 4128) = 716.23, p < 0.001; avoidance: F (2, 4128) = 403.24, p < 0.001), with a larger increase in reactance and avoidance compared to no label in the image-and-text HWL groups (p < 0.001) than in the text-only HWL groups (p < 0.001). There was a main effect of calorie information for avoidance (F (1, 4128) = 17.58, p < 0.001) and no main effect of calorie information for reactance (F (1, 4128) = 0.70, p = 0.405). Significant interactions between HWL group and calorie information (reactance: F (2, 4128) = 15.19, p < 0.001 avoidance: F (2, 4128) = 9.85, p < 0.001) indicated that calorie information increased scores for both reactance and avoidance compared to no label, decreased reactance but increased avoidance when it was added to text-only HWLs, and increased avoidance by a small amount but had no further impact on reactance in addition to image-and-text HWL.

#### 3.3.3 Perceived disease risk

Compared to the no label group, image-and-text and text-only HWLs increased perceived disease risk (all p < 0.001), but not calorie information alone (p = 0.28) (Table 3). The 3 × 2 ANOVA model indicated there was a main effect of HWL type (F (2, 4128) = 28.12, p < 0.001). In particular, there was a larger increase in perceived disease risk compared to no label in the image-and-text HWL groups (p < 0.001) than in the text-only HWL groups (p < 0.001). There was no main effect of calorie information (F (1, 4128) = 1.19, p = 0.276). There was no significant interaction between HWL group and calorie information (p = 0.464).

#### 3.3.4 Acceptability

A one-way ANOVA (F (4, 4129) = 134.36), with the text-only without calorie information group as the reference group, indicated that adding calorie information increased acceptability of the text-only HWL (p = 0.003). Image-and-text HWLs, with and without calorie information, were less acceptable than text-only HWLs (p = 0.017 and p < 0.001 respectively), with calorie information increasing acceptability of the image-and-text HWL. Calorie information alone was the most acceptable (p < 0.001).

## 4 Discussion

In an online selection task, all labels reduced energy-dense snack selection, supporting the study hypothesis. HWLs depicting the adverse health consequences of excess calorie consumption were more effective than calorie labels alone, with image-and-text HWLs being most effective compared to no label. In terms of secondary outcomes, all HWLs increased negative emotional arousal, reactance, avoidance and disease risk – with larger increases in the image-and-text HWL groups compared to text-only groups. Calorie information increased negative emotional arousal and avoidance, but not reactance and perceived disease risk. Text-only HWLs were more acceptable than image-and-text HWLs, but less acceptable than calorie information.

The current findings accord with previous findings concerning food products, indicating that presenting images of negative health outcomes encourage healthier selections (Rosenblatt et al., 2018a). The current findings also accord with findings for the use of HWLs on other harmful products. These show that image-and-text HWLs can decrease selection of SSBs (Mantzari et al., 2018) and that both text-only and image-and-text HWLs can increase the likelihood of smoking cessation behaviours and decrease alcohol selection, with image-and-text HWLs being most effective (Brewer et al., 2016; Clarke et al., 2020b; Hammond, 2011). The small effect of providing calorie information supports a recent review which concludes that calorie labelling may have a small effect on calorie intake but that it should form part of a wider set of measures to form a healthier food environment (Crockett et al., 2018). The findings of the current study suggest the effects of HWLs are potentially substantially larger and may overshadow the small impact of calorie labels.

Concerning the secondary outcomes, negative emotional arousal - fear, disgust, discomfort, worry - was increased in all label groups, but to a much larger degree in the HWL groups than observed with calorie labels, mirroring the pattern of label effectiveness. This may represent a key mechanism by which HWLs can impact behaviour, as also demonstrated in tobacco research (Brewer et al., 2019). This is in line with findings that indicated the potential mediating role of negative emotional arousal in online studies assessing the impact of HWLs on SSB selection (Mantzari et al., 2018) and intentions to consume SSBs (Donnelly et al., 2018). Relatedly, presenting aversive health-related images associated with snack foods, has been shown to make affective implicit cognitions more negative, mediating reduced preferences for those products (Hollands & Marteau, 2016). Strong negative emotions in response to HWLs have been found in other food and SSB studies (Grummon et al., 2019; Rosenblatt et al., 2019) and in tobacco (Cho et al., 2018), and alcohol (Clarke et al., 2020b) research, while calorie labels have previously been suggested to elicit negative emotions (Thunstrom, 2019) consistent with the small increases seen in the current study. Future research should formally test possible mediation of the impact of labels via negative emotional arousal using appropriate designs that allow causal relationships to be inferred.

Defensive reactions – reactance and avoidance – were demonstrated in response to all labels. These reactions were higher in response to HWLs compared to calorie information and were largest for image-and-text HWLs. This is in line with research showing defensive reactions to alcohol (Blackwell et al., 2018; Clarke et al., 2020b) and tobacco HWLs (Maynard et al., 2014; McCloud et al., 2017). In the current study, reactance was lower when calorie information was provided alongside a text-only HWL, but not when it was provided alongside an image-and-text HWL. This suggests that although it may not increase effectiveness, calorie information might still be important to provide in combination with health messages and could attenuate likely defensive reactions to HWLs. For avoidance, there were larger increases when calorie information was provided, which is in line with research indicating many participants ignore nutrition labelling unless they are searching for specific information (Ares et al., 2018). Avoidance does not necessarily reduce the potential benefit of a HWL; although negative emotions increase avoidance of tobacco HWLs, these negative emotions are also associated with cessation behaviours (McCloud et al., 2017). Avoidance has also been shown to be directly associated with quit attempts (Brewer et al., 2019) and behaviours that predict quit attempts (Hall, Mendel, Noar, & Brewer, 2018). The same may be true for reactance based on findings in the current study - image-and-text HWLs increased reactance more than text-only HWLs, but were more effective at reducing selection. However, these studies are often based on single-item subjective measures which may not be sufficient in assessing defensive reactions (Sillero-Rejon et al., 2018). Future studies should include both objective and subjective defensive reaction measures.

HWLs were less acceptable than calorie information only and image-and-text HWLs were less acceptable than text-only HWLs. Based on mean acceptability scores, the majority of HWLs (aside from image-and-text HWLs without calorie information) had a score higher than four (out of seven), which indicates participants viewed them as somewhat acceptable. Another recent study demonstrated similar acceptability ratings for image-and-text HWLs (Pechey et al., 2020). Due to the higher acceptability for text-only HWLs, and evidence of their effectiveness, it may be that HWLs in text form are the most promising labels for initial implementation. There was also some evidence that adding calorie information increased the acceptability of the HWLs. The acceptability of an intervention can increase as its perceived effectiveness increases (Reynolds et al., 2018) and support is generally higher for policies which are in further stage of their implementation (Bos, van der Lans, van Kleef, & van Trijp, 2018). Relatedly, HWLs may have a symbiotic effect with acceptability, where increased knowledge of health harms may increase acceptability (Watson, Weber, Hughes, Wellard, & Chapman, 2017) and the HWLs can increase the awareness of the links between calories, obesity and health harms – as demonstrated by perceived disease risk increases in the current study.

Beyond acceptability, another important factor to consider is the potential for HWLs to lead to unintended consequences, such as weight stigma, which can have a negative impact on health (Tomiyama et al., 2018). Research indicates that HWLs on SSBs can increase stigma (Hayward & Vartanian, 2019) and similar associations between stigma and ‘hard-hitting’ smoking campaigns have also been shown for those with smoking-related illnesses (Riley, Ulrich, Hamann, & Ostroff, 2017). Although the HWLs were designed in line with relevant guidance, further recommendations to minimise potential stigma include providing messages with counter-stigma themes alongside HWL messages (Hayward & Vartanian, 2019), *i.e.*, by emphasising the role of the food environment in obesity (Brandkvist et al., 2019). Relatedly, participants of all BMIs were included in the current study, but investigating differences in responses to HWLs by BMI may be an important avenue for future research.

### 4.1 Implications

The current findings indicate that image-and-text and text-only HWLs reduce selection of energy-dense snacks in an online hypothetical selection task, and are more effective than calorie information. These findings do not allow predictions of how people would react in real-life purchasing situations, therefore the impact of these HWLs – potentially in addition to calorie information - now merits investigation in more naturalistic settings using physical products and behavioural measures of selection and consumption. If effective, such labels could be a viable policy option for reducing the consumption of energy-dense snack foods and encouraging consumption of healthier products, although this should form one component of a broader policy strategy. Likely challenges from the food industry will be important to consider, potentially providing further support for initial introduction of text-only HWLs. It should be noted, however, that some countries are already moving towards warnings that more clearly communicate nutritional content, such as those recently implemented in Chile (Corvalán, Reyes, Garmendia, & Uauy, 2019).

### 4.2 Strengths and limitations

This large pre-registered online study in a general population sample is – to our knowledge - the only to date that looks at the impact of these HWLs, placed on energy-dense snacks, and their impact on food selection. It identifies the labels with the most potential for reducing energy-dense snack selection in a large, general population sample.

There are several limitations. First, the setting was artificial and the study task hypothetical. Replication is now needed in more naturalistic settings using behavioural measures of selection and consumption, where there is currently a gap in the evidence base (Clarke et al., 2020a). Second, the task measured immediate selection. Future studies should aim to assess the longer-term sustained impact of HWLs and calorie labels. Additionally, although for the purpose of this study energy density cut-offs based on categories from previous research were used (Pechey, Cartwright, et al., 2019; Pechey, Jenkins, Cartwright, & Marteau, 2019; Pechey & Marteau, 2018), the specific foods that might display HWLs warrants further consideration due to the complexities of considering single food items as part of a wider diet. Although many snack foods are high in energy, fat, sugar and low in nutrient content (Dunford & Popkin, 2017), and contribute to less healthy diets and obesity (WHO, 2018b), this is not true for all snack foods. For example, nuts are highly energy-dense but accepted as having substantial nutritional value. If such HWLs were to be implemented, nutritional scores might be taken into account alongside calories and energy-density, to enable a more complete perspective of the nutritional quality of the whole diet.

## 5 Conclusion

Health warning labels – particularly those that include an image and text - have the potential to reduce hypothetical selection of energy-dense snacks in an online setting. The impact of HWLs on selection and consumption in real-world settings using measures of actual selection and consumption awaits testing.

## Funding

This work was funded by a Collaborative Award in Science from 10.13039/100010269Wellcome Trust (Behaviour Change by Design: 206853/Z/17/Z to Theresa Marteau, Paul Fletcher, Gareth Hollands and Marcus Munafò).

The views expressed in this publication are those of the author(s) and not necessarily those of Wellcome Trust.

## Authors' contributions

GJH, TMM, NC, EP, EM and AKMB conceived the study and collaborated in designing the procedures. NC and EP coordinated the study and data collection. KD, MM and RKM performed the data analyses. NC and GJH drafted the manuscript, with all authors providing critical revisions. All authors read and approved the final manuscript.

## Declaration of competing interest

The authors declare that they have no competing interests.

## List of abbreviations

**CI**: Confidence Interval
**HWL**: Health Warning Label
**MD**: Mean Difference
**OR**: Odds Ratio
**SSB**: Sugar Sweetened Beverage
**TIPPME**: Typology of Interventions in Proximal Physical Micro-environments

## Availability of data and materials

The dataset supporting the conclusions of this article is available on the Open Science Framework project page: https://osf.io/k7tw5/ and the University of Cambridge Research Data Repository: https://doi.org/10.17863/CAM.54234.

## Footnotes

1 In our pre-registered protocol we included a further cognitive load manipulation selection task after the primary and secondary outcomes. Whilst we did conduct these analyses, we judged in line with the Editor and Reviewers that the results were not clearly interpretable as they did not include a manipulation check, therefore we opted not to include them in the manuscript. These results are available on the Open Science Framework (https://osf.io/7k46d/).
