# Supplementary material for: Impact of health warning labels on snack selection: An online experimental study
Source: Appetite. 2020 Nov 1;154:104744. doi: 10.1016/j.appet.2020.104744 (PMC7450271; doi:10.1016/j.appet.2020.104744)
Supplement: Multimedia component 1 [file mmc1.docx]

**Supplementary material**

**S1. Label development**

Label design

Large labels, with black lettering on a white background were used, based on tobacco packaging guidelines (1) and consistent with evidence that high contrasts between text and background enhance readability (2). Health consequences were based on existing evidence of the links between consuming excess calories, obesity and cancers, Type 2 diabetes and heart disease (3-5), as well as previous studies investigating similar labels or images on food products (6-8). We used more than one HWL based on recommendations from tobacco labelling to rotate the warnings that are used (1). Furthermore, this could increase engagement with the materials and maximise the likelihood that each participant viewing HWLs will be exposed to at least one that is impactful.

Label selection

The specific adverse health consequences used for the image-and-text HWLs used in the study were chosen based on the results of another study (9), which aimed to identify the images eliciting the highest levels of negative emotional arousal (see pre-registered protocol and analysis plan for this study: <https://osf.io/k7tw5/>). The study was conducted online using a between-subject design, in which 4618 participants were randomised to view one of 18 different image-and-text HWLs (see <https://osf.io/kf3r7/>) presented on an energy-dense snack (chocolate bar). These labels displayed an image depicting obesity or an adverse consequence of obesity, accompanied by a text statement stating that excess calories cause obesity and describing the adverse health consequence. The health consequences included bowel cancer, heart disease and general cancer (i.e., *excess calorie cause obesity, which causes 13 types of cancer*). Each label was presented and followed by outcome measures, to assess negative emotional arousal (a four-item measure), acceptability of the label and desire to consume the product, each rated on a 7-point scale. Selection of the three image-and-text HWLs for the main study was based primarily on mean negative emotional arousal scores, if scores were similar desire [to consume the product] scores were also taken into account. The following image-and-text health warning labels were selected:

1. *Adverse health consequence 1* (bowel cancer 1, Box S1*)*: an image of a diseased bowel alongside the caption “Excess calories cause obesity which causes bowel cancer” (negative emotional arousal score: M = 4.59, SD = 1.76; desire score: M = 3.05, SD = 1.90)
2. *Adverse health consequence 2* (Type 2 diabetes 1, Box S1*):* an image of a diseased eye alongside the caption “Excess calories cause obesity which causes Type 2 diabetes” (negative emotional arousal score: M = 3.85, SD = 1.72; desire score: M = 3.07, SD = 1.71)
3. *Adverse health consequence 3* (heart disease 1, Box S1): an image of heart surgery alongside the caption “Excess calories cause obesity which causes heart disease” (negative emotional arousal score: M = 4.03, SD = 1.70; desire score: M = 3.16, SD = 1.72)

These health consequences were used for all label groups. For the text-only group, the HWL did not display the image.

**References**

1. Hammond D. Tobacco Labelling & Packaging Toolkit: A guide to FCTC Article 11. 2009.

2. Vasiljevic M, Fuller G, Pilling M, Hollands GJ, Pechey R, Jebb SA, et al. What is the impact of increasing the prominence of calorie labelling? A stepped wedge randomised controlled pilot trial in worksite cafeterias. Appetite. 2019;141:104304.

3. Brown K, Rumgay H, Dunlop C, Ryan M, Quartly F, Cox A, et al. The fraction of cancer attributable to modifiable risk factors in England, Wales, Scotland, Northern Ireland, and the United Kingdom in 20152018.

4. Wang YC, McPherson K, Marsh T, Gortmaker SL, Brown M. Health and economic burden of the projected obesity trends in the USA and the UK. Lancet (London, England). 2011;378(9793):815-25.

5. WHO. Obesity and Overweight 2018 [Available from: <http://www.who.int/news-room/fact-sheets/detail/obesity-and-overweight>

6. Hollands GJ, Marteau TM. Pairing images of unhealthy and healthy foods with images of negative and positive health consequences: Impact on attitudes and food choice. Health Psychology. 2016;35(8):847-51.

7. Mantzari, Vasiljevic M, Turney I, Pilling M, Marteau T. Impact of warning labels on sugar-sweetened beverages on parental selection: An online experimental study. Preventive Medicine Reports. 2018;12:259-67.

8. Rosenblatt, Bode S, Dixon H, Murawski C, Summerell P, Ng A, et al. Health warnings promote healthier dietary decision making: Effects of positive versus negative message framing and graphic versus text-based warnings. Appetite. 2018a;127:280-8.

9. Pechey E, Clarke N, Mantzari E, Blackwell AK, De-Loyde K, Morris R, et al. Pictorial health warning labels on alcohol and food: potential effectiveness and acceptability Under Review.

**S2. Snack selection**

| **Snack (pack weight: grams)** | **Calories per pack** | **Calories per 100g** | **Number of participants selecting snack (% of total sample)** |
| --- | --- | --- | --- |
| **Energy-dense snacks** | | | |
| Doritos tangy cheese crisps (40g) | 203 | 510 | 260 (6%) |
| Gü zillionaire cheesecake dessert pot (91.5g) | 362 | 403 | 186 (5%) |
| Galaxy chocolate bar (42g) | 229 | 546 | 413 (10%) |
| Caramel shortcake slice (60g) | 320 | 533 | 150 (4%) |
| Dairy milk chocolate bar (45g) | 240 | 534 | 487 (12%) |
| McCoy’s salt and vinegar crisps (47.5g) | 251 | 529 | 424 (10%) |
| **Non energy-dense snacks** | | | |
| Tesco apple and grape snack pack (80g) | 45 | 57 | 285 (7%) |
| Nim’s beetroot and parsnip vegetable crisps (18g) | 59 | 328 | 480 (12%) |
| Tesco mango and pineapple fingers (80g) | 43 | 54 | 514 (12%) |
| Müller light toffee yoghurt (175g) | 99 | 51 | 256 (6%) |
| KIND dark chocolate and banana bar (38g) | 130 | 342 | 415 (10%) |
| Morrisons carrot crisps (20g) | 63 | 316 | 255 (6%) |

For snack images (see https://osf.io/kf3r7/)

**Table S3a. The interactions between HWL group and calorie information (present or absent) for each secondary outcome relating to Figure S3**

|  | Mean difference (95% CI), p value | | |
| --- | --- | --- | --- |
| Letters in reference to Figure A | **a** | **b** | **c** |
| **Negative emotional arousal** | 0.807 (0.633, 0.981) p < 0.001 | 0.132 (-0.43, 0.306)  p = 0.140 | 0.015 (-0.160, 0.189) p = 0.870 |
| **Reactance** | 0.478 (0.279, 0.676)  p < 0.001 | -0.299 (-0.498, -0.100)  p = 0.003 | -0.032 (-0.231, 0.166) p = 0.750 |
| **Avoidance** | 0.622 (0.420, 0.823) p < 0.001 | 0.056 (-0.146, 0.258)  p = 0.588 | 0.070 (-0.132, 0.271) p = 0.497 |
| **Perceived disease risk** | 0.277 (-0.226, 0.781)  p = 0.280 | 0.307 (-0.199, 0.813)  p = 0.234 | -0.098 (-0.602, 0.407) p = 0.704 |

a - represents the effect of calorie with no HWL present

b - represents the effect of calorie with text-only HWL present

c - represents the effect of calorie with image-and-text HWL present

**Table S3b. The mean differences for each recoded HWL group compared to no label group for each secondary outcome relating to Figure S3**

|  | **Recoded HWL group (mean difference (95% CI), p value)** | | |
| --- | --- | --- | --- |
|  | **Image-and-text (n = 1380)** | **Text-only (n = 1371)** | **No label (n = 1383)** |
| **Negative emotional arousal** | 2.16 (2.04, 2.28)  p < 0.001 | 1.48 (1.36, 1.60)  p < 0.001 | - |
| **Reactance** | 2.46 (2.32, 2.60)  p < 0.001 | 2.21 (2.07, 2.35)  p < 0.001 | - |
| **Avoidance** | 1.98 (1.84, 2.12)  p < 0.001 | 1.49 (1.35, 1.63)  p < 0.001 | - |
| **Perceived disease risk** | 1.32 (0.96, 1.68)  p < 0.001 | 0.96 (0.60, 1.31)  p < 0.001 |  |


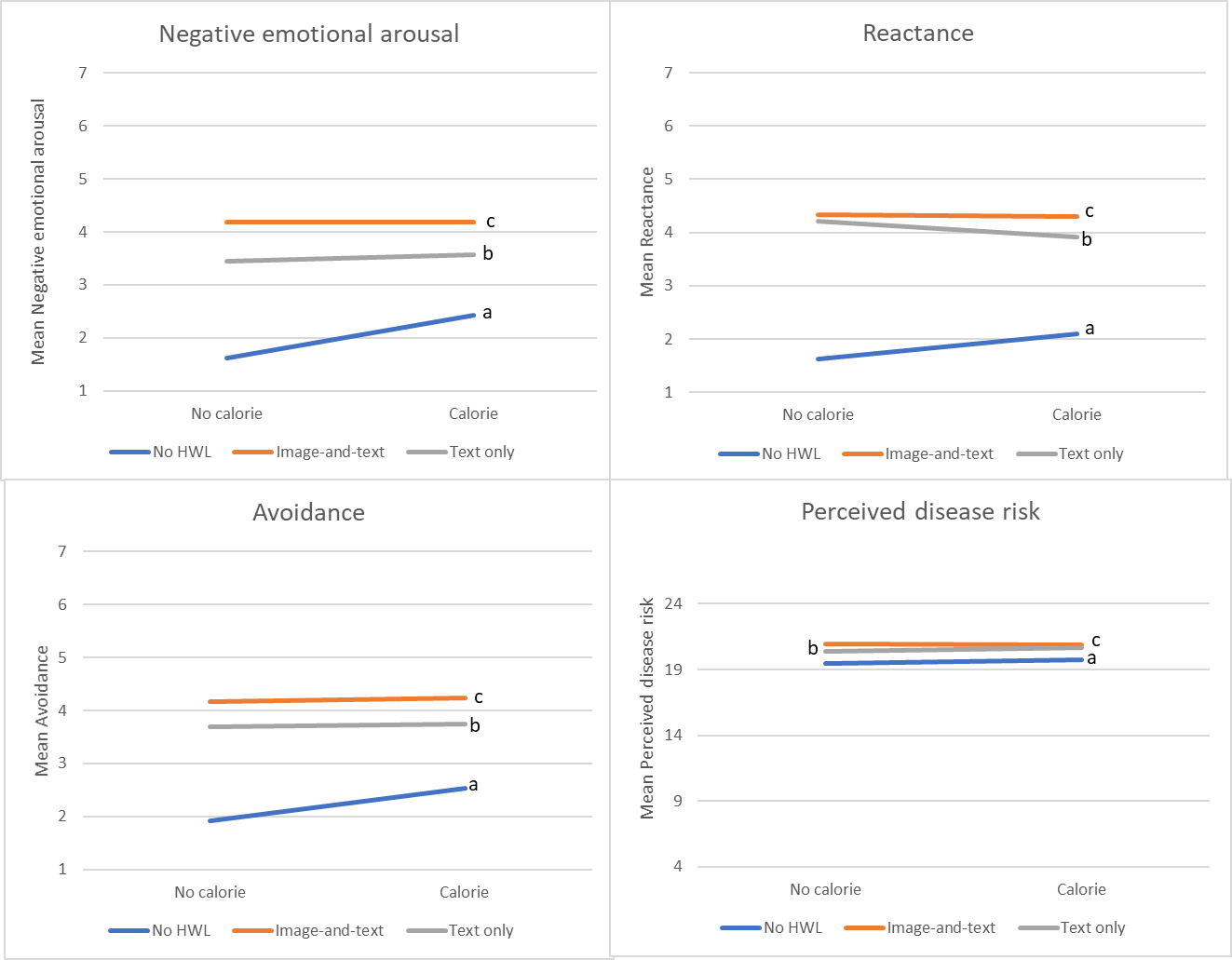
**Figure S3. Interactions between HWL group and calorie information for each secondary outcome.**

The no HWL line gradient (a) represents the effect of calorie with no HWL present. The text-only line gradient (b) represents the effect of calorie with text-only HWL present. The image-and-text line gradient (c) represents the effect of calorie with image-and-text HWL present.
